# Editorial: Determinants of Cell Size

**DOI:** 10.3389/fcell.2017.00115

**Published:** 2017-12-15

**Authors:** Mikael Björklund, Samuel Marguerat

**Affiliations:** ^1^Division of Cell and Developmental Biology, School of Life Sciences, University of Dundee, Dundee, United Kingdom; ^2^MRC London Institute of Medical Sciences, London, United Kingdom; ^3^Faculty of Medicine, Institute of Clinical Sciences, Imperial College London, London, United Kingdom

**Keywords:** cell size control, cell size regulation, growth rate, TOR Serine-Threonine Kinases, ion balance, cell volume, cell mass

Regulation of cell size has fascinated generations of biologists. While bacterial and eukaryotic cells maintain a characteristic size (Lloyd, 2013; Wood and Nurse, 2015; Westfall and Levin, 2017), cell size is not rigidly fixed and responds to external factors, particularly nutrient levels. This plasticity appears important for cell physiology and function under changing environments (Miettinen et al., 2017). Cellular mechanisms that control size homeostasis and plasticity may appear well defined and easy to generalize. However, surprisingly little is known about the exact nature of these processes and whether they require cells to “measure” or “know” their size. The intricate connection of cell size control with cell growth and division has been challenging for genetic approaches as other aspects of cell physiology may also be affected. Moreover, current evidence suggests that mechanisms of cell size control are complex, condition-specific, often redundant, and differ between organisms and cell types. The papers in “Determinants of Cell Size” research topic reflect this complexity and highlight the importance of integrated approaches in tackling the still elusive mechanisms that underlay cell size homeostasis both at steady state and in response to external stimuli (Shahrezaei and Marguerat, 2015).

Cell size at division is determined by the balance between cell growth (the increase in mass or volume) and the timing of cell division. Interestingly, faster growth rates in bacteria and eukaryotes lead to larger cell size. The mechanisms and functional relevance of this phenomenon remain unknown. In their mini-review Aldea et al. discuss this coordination and propose a “speedometer” model that can explain this phenomenon in the budding yeast *Saccharomyces cerevisiae*.

Advances in single cell biology and mathematical modeling are now also helping to understand cell size control. At the phenomenological level, many cell types follow the so-called “adder” principle (Campos et al., 2014; Taheri-Araghi et al., 2015). In this model cells “add” a constant volume (or mass) between cell divisions. Therefore, in relative terms, cells born large grow less than cells born smaller leading to size homeostasis at steady state. This elegant principle applies to many bacteria and eukaryotic cells, at least when growing under optimal conditions. However, cells do have the ability to deviate from the pure adder model. Priestman et al. show that *Mycobacterium smegmatis* cell size control is a robust adder under standard culture conditions, yet deviate from it on poorer carbon sources. Amir and co-workers similarly focus on how details matter in size control (Barber et al.). The authors use mathematical modeling to explore the coupling of cell size at division with cell cycle progression through either dilution of an inhibitor or accumulation of an activator protein that drives DNA replication initiation. Their work reveals that such mechanisms can explain size homeostasis in asymmetrically dividing budding yeast, but the sensitivity to noise make these accumulation/dilution models less likely to be at play in symmetrically dividing cells such as *E. coli*.

Target of rapamycin (TOR) signaling pathway is a major regulator of growth (mass accumulation) in animals and is of mainstream interest for the biological and biomedical communities due to its implication in aging, cancer, obesity, type 2 diabetes and neurodegeneration. The TOR pathway modulates cell size by sensing cellular metabolic status and external cues including nutrient levels, growth factors and stress. Gonzalez and Rallis review how TOR signaling affects growth through TORC1 and TORC2 signaling complexes that impact on temporal and spatial control of cell size and growth, respectively. Interestingly, they discuss that the functional separation of the two complexes may be less well defined than previously thought. The work by Tiedemann et al. provides a further example of how TOR pathway activity influences size, this time in osteoclasts, the monocyte-derived giant bone-degrading cells. Environmental conditions are once again a key determinant of cell size as culture media enriched with pyruvate stimulate osteoclast growth in TOR and AKT signaling dependent manner.

Increasing the number of macromolecules such as proteins, DNA and RNA is not the only way to modulate cell size. Cell volume regulation through ion balance is an extremely important mechanism providing osmotic stability. It is particularly relevant under acutely changing conditions in tissues such as kidney to regulate organismal water balance. The connection between cell size and osmotic balance can be explored with quantitative mathematical modeling of ion and water fluxes. As discussed by Kay in his review and tutorial, membrane potential resulting from gradients of monovalent inorganic ions such as Na^+^, K^+^, and Cl^−^ together with concentrations of impermeant intracellular molecules are critical determinants of cell size for all cell types. The report by Singh et al. complements this discussion on ion movements across plasma membrane. Studying the two known Na^+^K^+^2Cl^−^ (NKCC) co-transporters and their role in hyper-osmotic challenge and cell volume regulation, they show that NKCC isoforms have functional selectivity, with only NKCC1 functioning in cell volume recovery.

Together, the articles in this research topic highlight the importance of size control across model systems and provide examples of the mechanisms that lead to cell size homeostasis. Two main challenges remain: First, the molecular mechanisms that control cell size homeostasis have to be determined in greater detail. These are likely to vary from organism to organism, which is fascinating and illustrated by the breadth of model organisms used by the cell size community. Second, the functional and evolutionary constraints, if any, which dictate the preferred cell size in unicellular and multicellular organisms must be determined. In particular, we need to understand how cell size affects cellular functions as this may have far-reaching biomedical consequences as suggested by the disease associations of the TOR pathway. In summary, we believe that in years to come studies on cell size will substantially shape our understanding of normal physiology and pathologies ranging from infectious diseases to age-related metabolic diseases and hope this research topic will provide a taste of this.

## Author contributions

All authors listed have made a substantial, direct and intellectual contribution to the work, and approved it for publication.

### Conflict of interest statement

The authors declare that the research was conducted in the absence of any commercial or financial relationships that could be construed as a potential conflict of interest.

## References

[B1] Campos M. Surovtsev I. V. Kato S. Paintdakhi A. Beltran B. Ebmeier S. E.. (2014). A constant size extension drives bacterial cell size homeostasis. Cell 159, 1433–1446. 10.1016/j.cell.2014.11.022 25480302 PMC4258233

[B2] Lloyd A. C. (2013). The regulation of cell size. Cell 154, 1194–1205. 10.1016/j.cell.2013.08.053 24034244

[B3] Miettinen T. P. Caldez M. J. Kaldis P. Björklund M. (2017). Cell size control - a mechanism for maintaining fitness and function. Bioessays 39:1700058. 10.1002/bies.201700058 28752618

[B4] Shahrezaei V. Marguerat S. (2015). Connecting growth with gene expression: of noise and numbers. Curr. Opin. Microbiol. 25, 127–135. 10.1016/j.mib.2015.05.012 26093364

[B5] Taheri-Araghi S. Bradde S. Sauls J. T. Hill N. S. Levin P. A. Paulsson J.. (2015). Cell-size control and homeostasis in bacteria. Curr. Biol. 25, 385–391. 10.1016/j.cub.2014.12.009 25544609 PMC4323405

[B6] Westfall C. S. Levin P. A. (2017). Bacterial Cell Size: Multifactorial and Multifaceted. Annu. Rev. Microbiol. 71, 499–517. 10.1146/annurev-micro-090816-093803 28886685 PMC6018054

[B7] Wood E. Nurse P. (2015). Sizing up to divide: mitotic cell-size control in fission yeast. Annu. Rev. Cell Dev. Biol. 31, 11–29. 10.1146/annurev-cellbio-100814-125601 26566110

